# Indocyanine-Green Fluorescence-GUIDED Liver Resection of Metastasis from Squamous Cell Carcinoma Invading the Biliary Tree

**DOI:** 10.1155/2018/5849816

**Published:** 2018-06-10

**Authors:** Sara Benedicenti, Sarah Molfino, Marie Sophie Alfano, Beatrice Molteni, Paola Porsio, Nazario Portolani, Gian Luca Baiocchi

**Affiliations:** Department of Clinical and Experimental Sciences, Surgical Clinic, University of Brescia, Brescia, Italy

## Abstract

*Background. *The concept of fluorescence-guided navigation surgery based on indocyanine green (ICG) is a developing interest in many fields of surgical oncology. The technique seems to be promising also during hepatic resection.* Case Presentation. *We reported our experience of ICG-fluorescence-guided liver resection of metastasis located at VIII Couinaud's segment from colon squamous cell carcinoma of a 74-year-old male patient.* Results. *After laparotomy, the fluorescing tumour has been clearly identified on the liver surface. We have also identified that a large area of fluorescent parenchyma that gets from the peripheral of the lesion up to the portal pedicle such as the neoplasia would interest the right biliary tree in the form of neoplastic lymphangitis. This datum was not preoperatively known.* Conclusion. *Fluorescent imaging navigation liver resection could be a feasible and safe technique helpful in identifying additional characteristics of lesion. It could be a powerful tool but further studies are required.

## 1. Introduction

Indocyanine green (ICG) is a water-soluble, anionic, amphiphilic tricarbocyanine molecule that emits fluorescence upon illumination with near infrared light [1, 2].

For several years, it has been used in ophthalmic angiography and for determining cardiac output and hepatic function [3]. However, it has only recently showed practicability and feasibility in the field of surgical oncology [4–9].

Focusing on liver metastases, many studies showed how near infrared fluorescence imaging using indocyanine green is a promising technique to intraoperatively visualize the contrast between hepatic lesions and normal liver tissue in real time and how it could be considered a powerful tool to help surgery in the “fluorescent-guided surgery” [10–12].

Referring to the surgical treatment of colon cancer metastatic disease, liver resection is allowed in about 20% of patients [13]. The problem of getting a complete removal of tumour is the presence of residual foci of tumour cells, associated with tumour recurrence and lower survival rates.

In this case the “ICG-fluorescence-based navigation” can be a useful tool in order to locate supplementary lesions that could have been missed with current imaging techniques, such as computer tomography (CT), magnetic resonance imaging, and ultrasonography (US) [12].

Based on recent studies that suggest the effective utility of Photodynamic-Eye (PDE) assessment of ICG fluorescence (PDE + ICG) combined with Intraoperative Ultrasound (IOUS) for ensuring the complete surgical removal of liver metastases from colorectal cancer [14–16], we report our experience with the use of this technique during resection of liver metastasis from colon squamous cell carcinoma, invading the biliary tree.

## 2. Case Report

**Figure 1:**
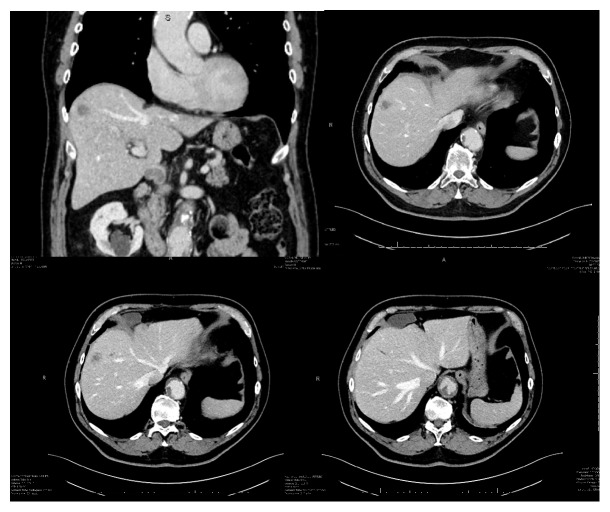
Preoperative CT imaging of hepatic lesion.

A 74-year-old male patient was admitted to our hospital in March 2017 to undergo liver resection to treat a malignant hepatic lesion diagnosed with CT and PET and a fine-needle biopsy positive for squamous carcinoma. The hepatic tumour discovered during follow-up for a previous bladder cancer submitted to endoscopic surgery three years before measured 22 mm in diameter and was located in the VIII Couinaud's segment [17] of the liver in association with three smaller hypodense liver lesions with a focal dilatation of peripheral biliary tree (Figure 1).

The case is discussed with radiologists, oncologists, and pathologists of our hospital. Even if the lesion had been the single site of disease; due to the proximity/doubtful infiltration of the lesion to the biliary tree, we decided to submit the patient to an explorative staging laparotomy and possible palliative surgery.

Our internal protocol states that during the preadmission every patient who is a candidate for a liver resection is subjected to a routine liver function test with ICG to determinate the most appropriate surgical procedures [18]: 0,5 mg/Kg ICG are routinely injected intravenously up to seven days before surgery to evaluate the ICG retention rate at 15 min (R15). In our case 45 mg of ICG was intravenously administrated to test hepatic function, ten days before the surgery (patient R15 = 8.9).

Thanks to the ICG property of being fluorescent with the light emitted from the photodynamic eye of the laparoscopic system in our possession, it is possible to visualize the lesion during the surgical procedure. To this target, timing of administration and dose of ICG are key points.

**Figure 2:**
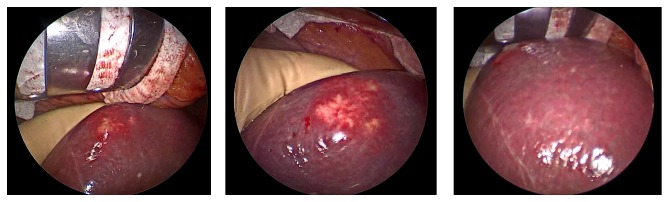
Intraoperative visualization of metastatic lesion at the liver surface.

**Figure 3:**
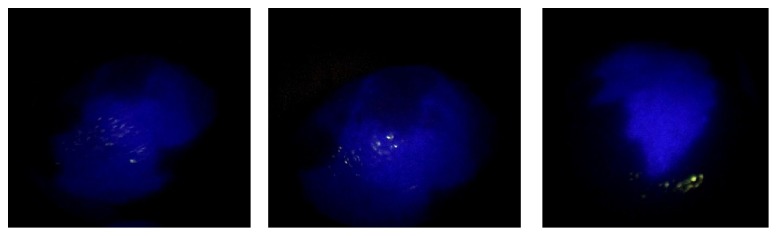
Intraoperative visualization with ICG-PDE system: the small lesion with rim staining around the tumour and the fluorescence emitted region from the cholestatic area.

Several studies have demonstrated that the effective dose of ICG depends on the timing of injection; in particular, if the function liver test had been performed more than 7 days before surgery it would have been necessary to administer an adjunctive dose (0,1 mg/Kg) the day before [10]. In this case, it was necessary to administrate an adjunctive dose of ICG the day before the surgery (9 mg of ICG injected intravenously). After laparotomy, exploration of the abdominal cavity, and exposure of the liver, we easily confirmed the superficial lesion in the VIII Couinaud's segment. The liver surface has been analysed with the fluorescent imaging system. The fluorescing tumour has been clearly identified and defined on the liver surface, as shown in Figure 2. We have also identified that a large area of fluorescent parenchyma that gets from the peripheral of the lesion up to the portal pedicle such as the neoplasia would interest the right biliary tree in the form of neoplastic lymphangitis (Figure 3). This datum was not preoperatively known.

A right hepatectomy would have been the oncologically correct surgical procedure due to the infiltration of right biliary duct. Considering the probable metastatic nature of the lesion, the absence of a clearly primary lesion, the age, the comorbidities, and the small size of residual liver, we have decided to perform an atypical segmental resection of S8 associated with cholecystectomy and lymphadenectomy of the hepatic pedicle nodes, including the area of impaired biliary excretion.

At the histological examination, the lesion, the lymph nodes of the hepatic pedicle region, and the right biliary branch, respectively, resulted in hepatic metastases from squamous cell carcinoma and sites of metastatic location. As expected the resection margin was interested by neoplasia.

In particular, the histological examination showed the following:

- Macroscopical exam: the neoplasia, in a site, appears to be in contact with the resection margin
- Microscopical exam: parenchymal hepatic section that showed metastatic localization of squamous carcinoma moderately differentiated. The neoplasia interest the surgery resection margin.

**Figure 4:**
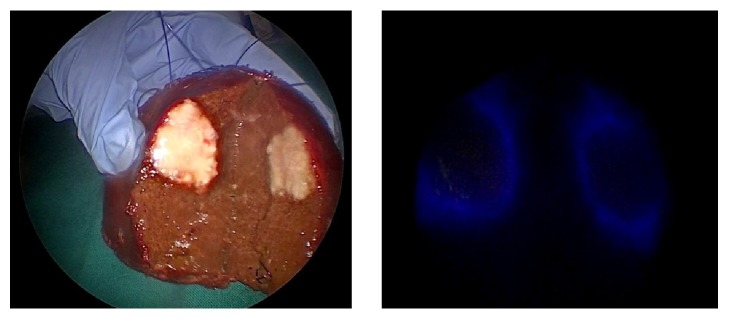
Normal and fluorescent pattern of the tumour in the resected specimen (rim fluorescence type).

**Figure 5:**
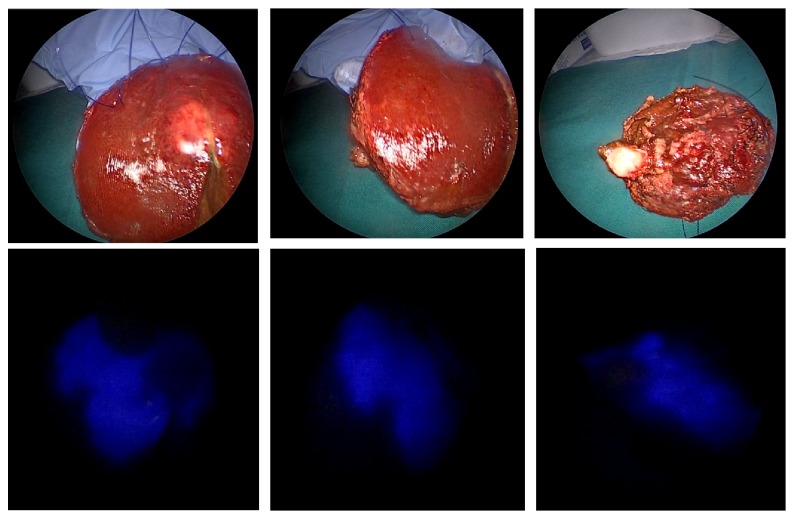
Visualization of cholestatic area by fluorescence on the cut surface.

In this case, fluorescent imaging has revealed a fluorescing ring around the hepatic metastasis (Figure 4). The fluorescence of the cholestatic area was shown on the cut surface (Figure 5).

## 3. Discussion

ICG-fluorescent imaging techniques have begun to be used in hepatobiliary procedures in the last few years. These techniques are expected to complement conventional imaging techniques without having the purpose to replace all of the roles of conventional techniques. It provides simultaneous, real time, and high resolution identification of many type of different structures and, particularly during the navigation surgery application, could be a helpful tool to discover adjunctive lesions and identify segmental staining and biliary tree visualization [9, 11, 15, 19, 20]. 

The major advantage of this system is that it overlays fluorescent images on the background colour images. This property can make it easier for a surgeon to understand the exact location of the fluorescing lesions (liver cancer/or bile ducts) on the surrounding structures as compared with that in the conventional fluorescent imaging system [16, 21].

The fluorescent imaging of segmental cholestasis region, caused by the duct tumour invasion or thrombi and demonstrated at the intraoperative PDE-visualization, could be explained by the fact that ICG has been retained in the noncancerous liver parenchyma due to bile ducts obstruction and the consecutive altered biliary excretion in this tissue [22].

Our case report shows how feasibility and safety of this procedure could help to identify additional characteristics of the lesion, such as the cholestasis area around the lesion itself and infiltration of the biliary tree too with minimal adjunctive costs [23–25]. So it could be simple and helpful to guide a surgical procedure.

## 4. Conclusion 

The major advantages of fluorescent imaging are its feasibility and safety. Once ICG is preoperatively administered for liver function testing, we can obtain fluorescent images of tumour and cholestasis regions in real time by simply placing the camera imaging on the liver surface before the resection, during the liver resection, and over surgical specimens.

Further and ongoing studies are required to better define the feasibility and usefulness of this technique.
